# Extracellular vesicles from the apoplastic fungal wheat pathogen *Zymoseptoria tritici*

**DOI:** 10.1186/s40694-020-00103-2

**Published:** 2020-09-18

**Authors:** Erin H. Hill, Peter S. Solomon

**Affiliations:** Division of Plant Sciences, Research School of Biology, The Australian National University, Canberra, 2601 Australia

**Keywords:** Extracellular vesicles, *Zymoseptoria tritici*, Wheat disease

## Abstract

**Background:**

The fungal pathogen *Zymoseptoria tritici* is a significant constraint to wheat production in temperate cropping regions around the world. Despite its agronomic impacts, the mechanisms allowing the pathogen to asymptomatically invade and grow in the apoplast of wheat leaves before causing extensive host cell death remain elusive. Given recent evidence of extracellular vesicles (EVs)—secreted, membrane-bound nanoparticles containing molecular cargo—being implicated in extracellular communication between plants and fungal pathogen, we have initiated an in vitro investigation of EVs from this apoplastic fungal wheat pathogen. We aimed to isolate EVs from *Z. tritici* broth cultures and examine their protein composition in relation to the soluble protein in the culture filtrate and to existing fungal EV proteomes.

**Results:**

*Zymoseptoria tritici* EVs were isolated from broth culture filtrates using differential ultracentrifugation (DUC) and examined with transmission electron microscopy (TEM) and nanoparticle tracking analysis (NTA). *Z. tritici* EVs were observed as a heterogeneous population of particles, with most between 50 and 250 nm. These particles were found in abundance in the culture filtrates of viable *Z. tritici* cultures, but not heat-killed cultures incubated for an equivalent time and of comparable biomass. Bottom-up proteomic analysis using LC–MS/MS, followed by stringent filtering revealed 240 *Z. tritici* EV proteins. These proteins were distinct from soluble proteins identified in *Z. tritici* culture filtrates, but were similar to proteins identified in EVs from other fungi, based on sequence similarity analyses. Notably, a putative marker protein recently identified in *Candida albicans* EVs was also consistently detected in *Z. tritici* EVs.

**Conclusion:**

We have shown EVs can be isolated from the devastating fungal wheat pathogen *Z. tritici* and are similar to protein composition to previously characterised fungal EVs. EVs from human pathogenic fungi are implicated in virulence, but the role of EVs in the interaction of phytopathogenic fungi and their hosts is unknown. These in vitro analyses provide a basis for expanding investigations of *Z. tritici* EVs *in planta,* to examine their involvement in the infection process of this apoplastic wheat pathogen and more broadly, advance understanding of noncanonical secretion in filamentous plant pathogens.

## Background

*Zymoseptoria tritici* is the fungal pathogen responsible for Septoria Tritici Blotch (STB) of wheat. This disease threatens wheat production in the world’s temperate cropping regions, causing particularly significant yield reductions across Europe [1, 2]. STB is a foliar disease, beginning with the germination of *Z. tritici* spores on the leaf surface and hyphal entry into the host tissue via stomata. On penetration, *Z. tritici* hyphae slowly colonise the extracellular spaces of the mesophyll over a long latent period of approximately 8–11 days, depending on the pathogen isolate and wheat cultivar combination [3, 4]. The pathogen then transitions to necrotrophic growth: host cell death is induced, manifesting as necrotic lesions on the leaves and releasing nutrients to fuel fungal growth. This allows the pathogen to produce mature asexual fruiting bodies, pycnidia, that disseminate pycnidiospores and spread infections to adjacent leaves and neighbouring plants [3].

The latent period of *Z. tritici* infection is symptomless and the pathogen is suggested to gain nutrients from its own lipid stores, rather than exploiting host nutrients [5]. At this point, host defences are not strongly induced, putatively due to pathogen evasion or suppression, until the switch to necrotrophy when there is a ‘hyperactivation’ of defence responses [5]. The molecular events occurring during this latent period and necrotrophic switch remain unclear but it is likely effectors—in the form of proteins or secondary metabolites (SM) and potentially small RNAs (sRNAs)—are secreted to the apoplast and/or adjacent wheat cells to suppress or manipulate host defence responses [5, 6]. Significant genomic, transcriptomic and functional work has aimed to identify and characterise these *Z. tritici* pathogenicity factors, with many studies focusing on proteins with canonical N-terminal secretion signals (or signal peptides (SPs)) that are putatively exported from the cell via the ER-Golgi pathway [5, 7–11]. Despite these efforts, few secreted effector proteins have been shown to contribute to the disease phenotype, likely partly due to the functional redundancy in effector proteins and the quantitative nature of host resistance/pathogen virulence in the wheat—*Z. tritici* interaction [5, 12–14]. It is arguable that in filtering proteins for canonical SPs in these studies, a subset of effectors or other virulence factors could have been missed, as effectors lacking SPs have been characterised in at least three fungal phytopathogens [15, 16]. It is also important to recognise that even some proteins with canonical SPs are likely secreted via non-canonical pathways, as shown for effectors in the oomycete *Phytophthora infestans* and the fungal rice pathogen *Magnaporthe oryzae* [17, 18]. Given this and the fact that a proportion of the experimentally-defined *Z. tritici* secretome lacks SPs, it is possible non-canonical pathways may play a role in the secretion of *Z. tritici* virulence factors [19, 20]. To date, the role of these non-canonical secretory pathways in the *Z. tritici*—wheat interaction is unexplored.

Extracellular vesicles (EVs) are a non-canonical secretion mechanism implicated in cell–cell communication in and between organisms [21]. These vesicles are lipid bilayer-bound structures that carry molecular cargo, including proteins, lipids and nucleic acids. They are secreted from cells to the extracellular space as a heterogeneous population, varying in size, cargo and biogenesis [22]. Recent evidence from plants suggests EVs may be involved in cross-kingdom communication between plants and their microbial pathogens [23, 24]. *Arabidopsis thaliana* releases EVs to the apoplast that are enriched in defence and stress-associated proteins and ‘tiny’ small RNAs (sRNAs). These EVs are hypothesised to be involved in defence against the bacterial pathogen *Pseudomonas syringae* [25, 26]. *Arabidopsis thaliana* has also been suggested to secrete EVs carrying sRNAs involved in host-induced gene silencing of genes in the fungal pathogen *Botrytis cinerea* [23], while EVs from sunflower leaves were implicated in inhibiting the germination and growth of *Sclerotinia sclerotiorum* ascospores [27].

Fungi also secrete EVs but their role in plant–pathogen interactions has yet to be examined. Fungal EV research has largely focused on *Saccharomyces cerevisiae* and the human pathogens *Candida albicans, Cryptococcus neoformans* and *Histoplasma capsulatum* (recently reviewed in Bielska and May, Bleakley et al. and Silva et al. [28–30]); this has led to the description of EVs from these pathogen as ‘fungal virulence bags’ [31]. To date, EVs have only been isolated from one plant pathogenic fungus, the cotton wilt pathogen *Fusarium oxysporum* f. sp. *vasinfectum* (*Fov*). *Fov* EVs are phytotoxic on cotton and *Nicotiana benthamiana*, but their role in the infection lifecycle of the pathogen is unknown [32].

Given the apoplastic lifestyle of *Z. tritici*, we speculated that EVs may be secreted by the pathogen during its colonisation of the apoplast, perhaps to secrete pathogenicity factors or target molecules to neighbouring host cells. To pave the way for these investigations, we aimed to isolate *Z. tritici* EVs produced in vitro and perform preliminary proteomic profiling of *Z. tritici* EV protein cargo. These insights into the secretion and cargo of *Z. tritici* EVs provide a foundation for expanding these investigations *in planta* and assessing the role of *Z. tritici* EVs in the apoplast during infection of wheat.

## Results

### EVs can be isolated from *Z. tritici* cultures

**Fig. 1:**
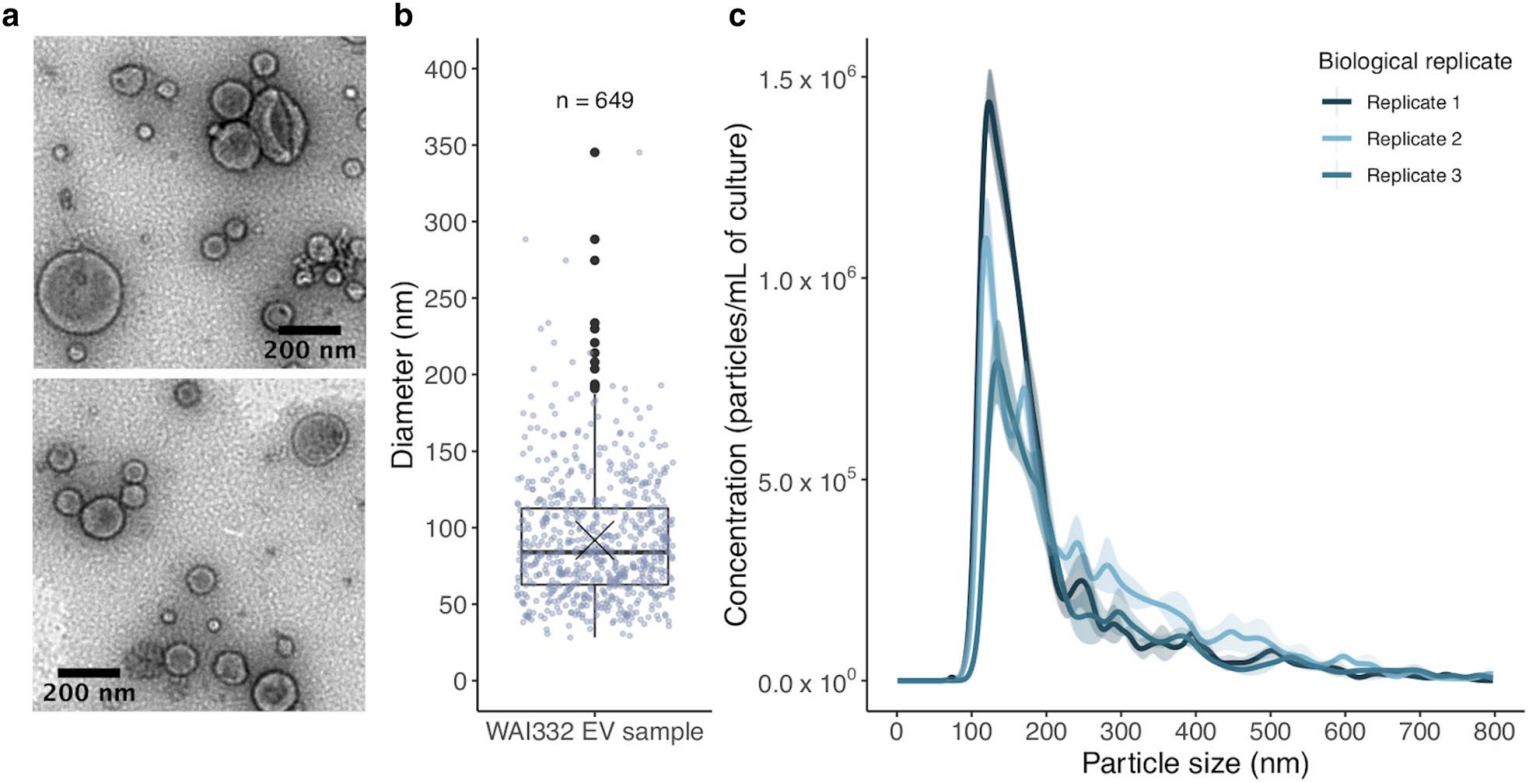
A heterogeneous population of EVs is secreted by *Z. tritici* in vitro. **a**, **b** are representative of several biological replicate EV samples, with (**a**) showing representative TEM data of 2% uranyl acetate-stained EV samples. Images were cropped and scale bars added using ImageJ; no other modifications were made. **b** Boxplot showing the distribution in EV diameter, measured using ImageJ of the representative EV sample, shown in **a**. The median particle size was 84.0 nm; the cross represents a mean particle size of (91.83 nm ± 39.9 sd). Each point on the boxplot represents an EV-like particle from the TEM data (n = 649). **c** NTA data showing particle size versus concentration of particles per mL of fungal culture for 3 biological replicates. The size distributions were averaged across 3 technical replicates and the standard error for each plotted value is indicated with shading. Mean particle size ranged from 168.0–195.2 nm and the modal particle size from 134.13 to 150.3 nm

To determine if *Z. tritici* secretes EVs under in vitro culture conditions, as described for other fungi, we isolated EVs from *Z. tritici* broth cultures using a differential ultracentrifugation (DUC) method adapted from Rodrigues et al. [59] and Thery et al. [60]. We observed particles resembling fungal and mammalian EVs, based on their comparable morphology and size distribution, using transmission electron microscopy (TEM) and nanoparticle tracking analysis (NTA) (Fig. 1). TEM analysis revealed a heterogeneous population of EVs ranging from < 50 to > 300 nm in size, with most particles falling between 50—150 nm and median and mean particle size of 84.0 nm and 91.8 nm (sd 39.9 nm), respectively. Some particles were > 300 nm but these were less frequently observed (Fig. 1a, b). NTA corroborated the TEM-derived EV population size distributions, revealing a dominant peak in the particle size distribution between approximately 100–250 nm (Fig. 1c). The apparent lack of smaller particles represented in the NTA size distribution when compared to the TEM measurements, may be artefactual of NTA systems’ known underreporting of particles < 50 nm and/or the dehydration and shrinkage caused by negatively staining EVs for TEM [21, 33]. *Z. tritici* EV samples typically had a concentration of 0.12–0.15 µg of protein and 1.45–2.43 × 10^8^ particles per mL of culture filtrate. While *Z. tritici* isolate WAI332 grown in the semi-defined medium Fries 3 was typically used for EV isolations, we found that EVs could also be isolated from the same isolate cultured in potato dextrose broth and minimal media and from another Australian isolate, WAI321 (Additional file 1: Figure S1).

**Fig. 2:**
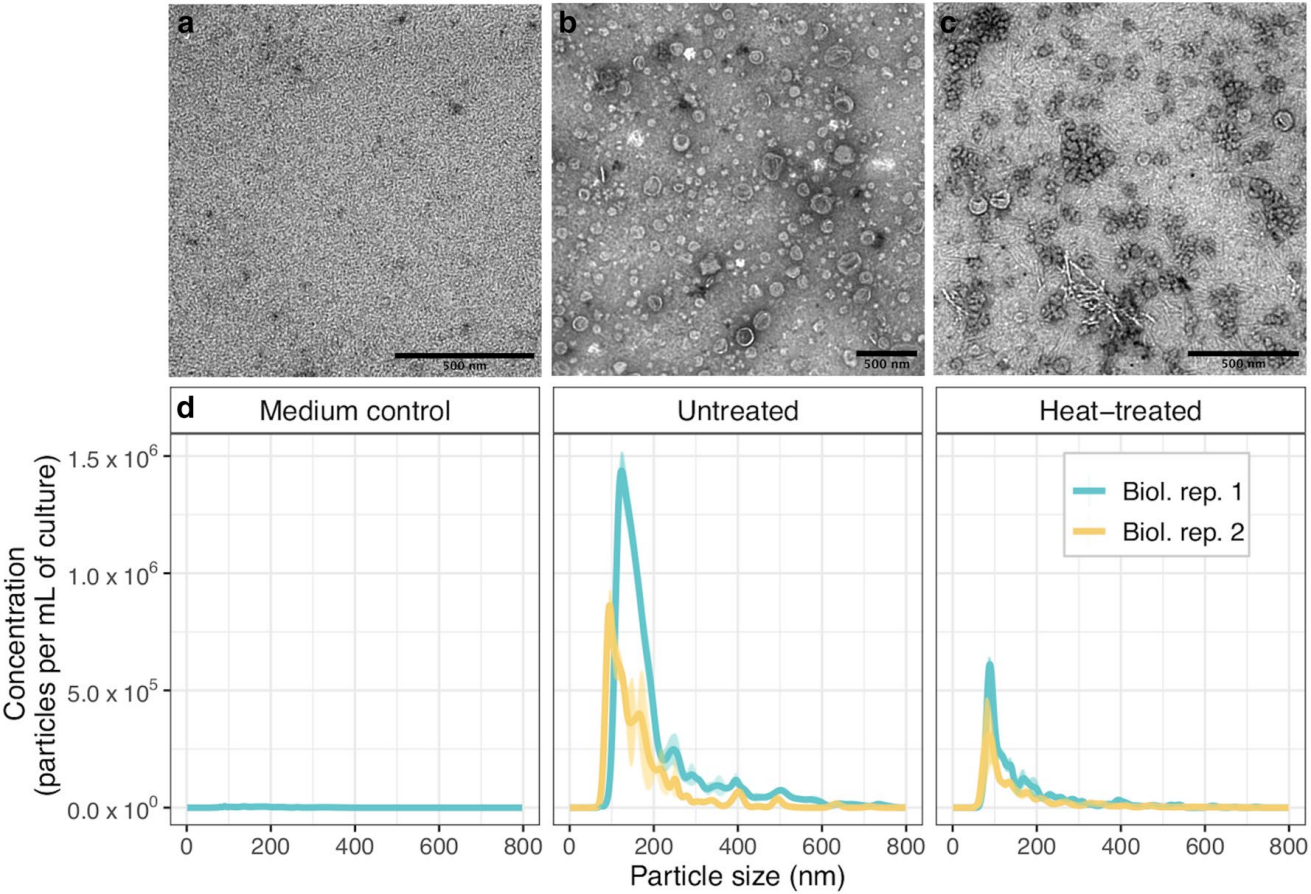
EVs are abundantly produced by viable *Z. tritici* cultures but not heat-treated cultures. **a**–**c** TEM of samples prepared using differential ultracentrifugation from a medium-only control culture, untreated and heat-treated *Z. tritici* cultures, respectively. Scale bars represent 500 nm. **d** Nanoparticle tracking analysis of the corresponding samples: plots represent the mean particle concentration per mL of culture filtrate versus particle size (nm), with each size distribution averaged over three technical replicates. The mean and modal particle size for the untreated condition ranged from 135.7–168.0 nm and 109.7–134.1 nm, respectively. Mean and modal size of particles in the heat-treated condition were 125.1–129.3 nm and 93.0–102.5 nm, respectively. Shading along the size distributions represents the standard error of each plotted value

EVs are described as membrane-bound, cargo-containing particles released by cells. We wanted to confirm the EVs observed in *Z. tritici* cultures were the product of viable cells rather than membranous artefacts of senescing cells or cell debris. *Z. tritici* is a heat-labile fungus so we compared the presence of EVs in heat-treated cultures with that of untreated cultures. *Z. tritici* cells were heat-treated after 72 h of growth and incubated in fresh culture medium alongside viable cultures for a further 72 h, until the latter were of equivalent biomass (Fig. 2, Additional file 2: Table S1). Heat-treated cells were not viable when plated on YSA (data not shown). TEM suggested heat-treated cultures produced samples rich in irregularly-shaped debris, with some EV-like particles observed (Fig. 2b, c). Comparatively, EVs were abundant in the non-heat-treated culture. Nanoparticle tracking analysis indicated particles in the 100–250 nm range were less concentrated in heat-treated cultures. EVs from untreated cultures had mean and modal sizes ranging from 135.7–168.0 nm and 109.7–134.1 nm from two biological replicates, respectively. Particles from heat-treated cultures ranged from 125.1–129.3 nm in average size and 93.0–102.5 nm in modal size (Fig. 2d; Additional file 1: Figure S2). We also found EV-like particles were absent from the medium only control, confirming EVs were not an artefact of the semi-defined growth medium or isolation procedure (Fig. 2a, d).

### *Z. tritici* EVs have a protein profile distinct from soluble secreted proteins in the broth culture filtrate

It is well established that EVs from mammals, fungi, plants and bacteria contain protein cargo so we aimed to profile the protein content of *Z. tritici* EVs released under in vitro growth conditions using bottom-up proteomics. From five biological replicate EV samples we identified 240 high-confidence (HC) proteins consistently detected using LC–MS/MS; these proteins were identified using a minimum of two unique peptides in at least 4/5 EV replicates (Table 1). The full list of proteins is provided in Additional file 2, Table S2. We made qualitative presence/absence comparisons of protein content in EV samples with soluble secreted proteins (SS); the SS fraction was prepared from the supernatant separated from EVs by UC at 100,000×*g*. This supernatant was again centrifuged at 120,000×*g* to remove remaining EVs and insoluble debris. LC–MS/MS analysis of the SS fraction identified 79 HC proteins (detected in at least 2/3 biological replicates).

**Table 1:** Characteristics of proteins identified in *Z. tritici* EV samples and the soluble secreted fraction

Proteins identified using LC–MS/MS	EV proteins	Soluble secreted proteins (SS)
Biological replicates	5	3
Total proteins identified	771	99
High confidence (HC) proteins^1^	240	79
*HC proteins unique to sample type*^2^	*210 (100%)*	*49 (100%)*
Proteins with signal peptides	14 (6.7%)	38 (77.6%)
Proteins with transmembrane domains/GPI-anchors^3^	15 (7.1%)	1 (2.0%)
Putative Cazymes	7 (3.3%)	6 (12.2%)
Putative proteolytic enzymes	6 (2.5%)	3 (6.1%)
Predicted effectors^4^	17 (8.1%)	16 (32.7%)
Median protein size	426 aa	257 aa

^1^Proteins were high-confidence if present in ≥ 4/5 EV biological replicates or ≥ 2/3 SS replicates, with ≥ 2 unique peptides detected

^2^Protein counts and percentages in the following rows are in reference to the subset of HC proteins detected only in EVs *or* SS fractions; proteins detected in both fractions were excluded from these counts

^3^Transmembrane domains predictions by TMHMM were included when more than one domain predicted, or if only one was predicted, this was not in the first 60 amino acids

^4^Effector count includes proteins predicted as effector by both v1.0 and v2.0 of EffectorP

**Fig. 3:**
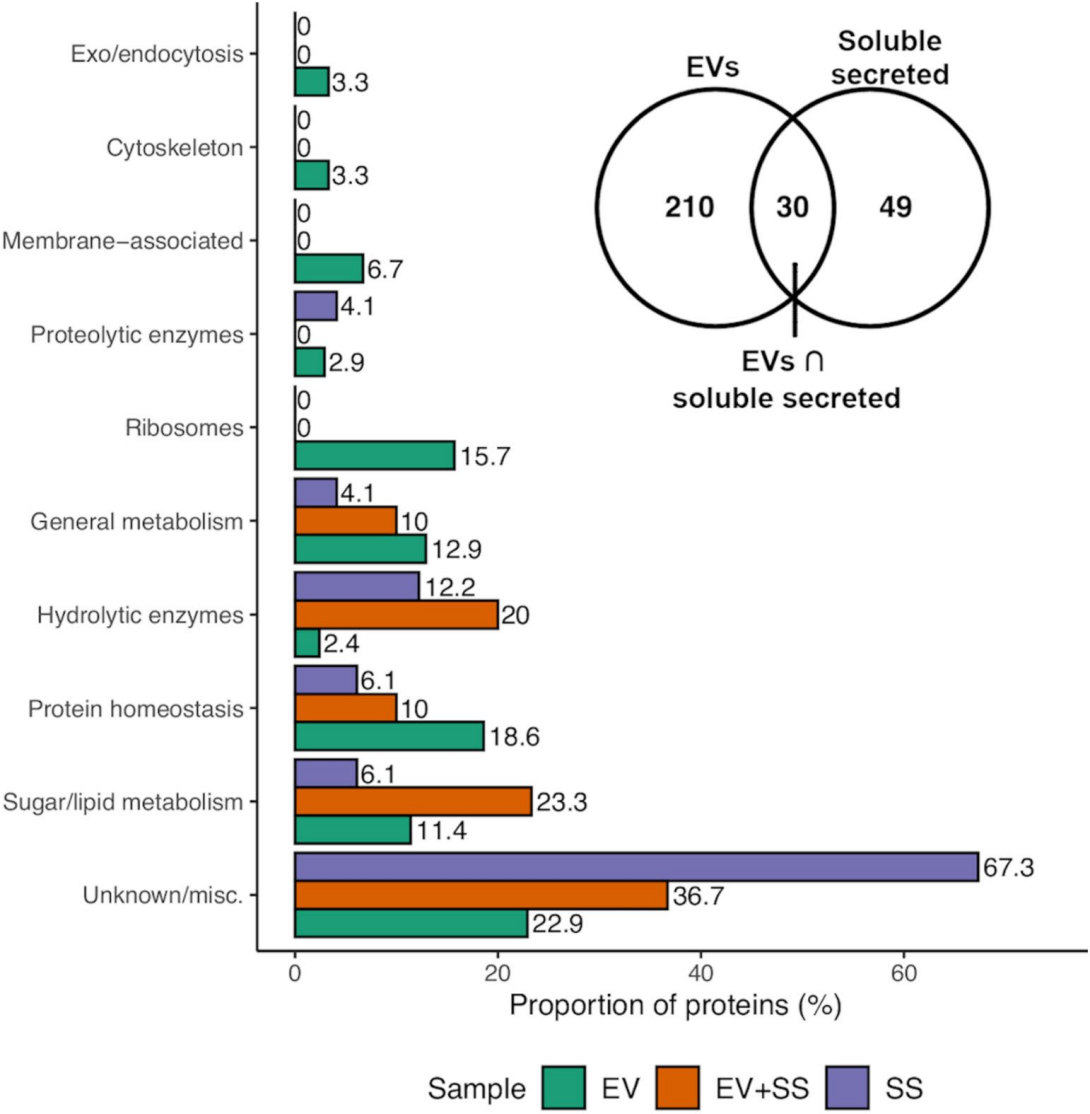
Functional classification of EV and soluble secreted (SS) proteins. Proteins were manually classified according to sequence similarity to proteins in the Uniprot database and the presence of conserved protein domains. The inset venn diagram shows the overlap of the EV and SS protein sets compared in the bar chart; proteins identified in both EV and SS fractions, denoted EV + SS, are also represented in the bar chart

Of the 240 HC EV proteins, 210 were present only in the EV samples, while 49 SS proteins were unique to SS samples and 30 proteins were shared between the fractions (Fig. 3a, Additional file 2: Table S3). Whether the presence of proteins in both fractions is due to insufficient purification of EVs, contamination from disrupted EVs, or the secretion of proteins via EVs and canonical secretion pathways will require further investigation. Given this, we have focused on HC proteins unique to either the EV or SS fractions; values reported will be in reference to these HC, fraction-specific protein groups.

To probe potential differences in the characteristics of proteins in the EV and SS fractions we identified those with canonical secretion signals (SPs) and transmembrane domains (TMDs) or glycosylphosphatidylinositol-(GPI) anchors, the latter being features of membrane-associated proteins (Table 1; Additional file 2: Table S2, S3). The proportion of proteins with canonical SPs was greater (77.6%) in the HC SS fraction than in EVs (6.7%). The presence of proteins without a SP in yeast and filamentous fungal culture filtrates has been reported and may result from non-canonical secretion mechanisms [15, 34, 35]. Proteins lacking SPs have been identified in proteomic analyses of both *in planta* and in vitro *Z. tritici* secretomes [19, 20, 36]. EV proteins largely lacked SPs, aligning with previous analyses of fungal EV protein cargo [37]. EVs had more predicted membrane-associated proteins (7.1%) compared to the secreted fraction (2.0%).

We also aimed to identify proteins potentially important for virulence, such as hydrolytic enzymes like cell wall degrading enzymes (CWDEs), proteases and effector proteins, in EV and SS fractions. The gene and protein expression profiles of these virulence-associated proteins induced in vitro may not reflect their expression during *Z. tritici in planta* infection. However, some in vitro growth conditions, such as Fries medium, can induce the expression of virulence-associated genes, including effectors and secondary metabolites, of plant pathogenic fungi [38, 39]. We found that hydrolytic enzymes—including proteins with carbohydrate active enzymatic domains (CAzymes) or peptidase protein domains—constituted a larger proportion of SS proteins (12.2% and 6.1%, respectively) compared to EV proteins (3.3% and 2.5%). Similarly, more SS proteins were predicted to be effectors by EffectorP (32.7% *vs* 8.1% in EVs) (Table 1, Additional file 2: Table S4). Putative effectors in SS samples were largely uncharacterised proteins. In comparison, EV-associated predicted effectors often had high sequence similarity to characterised proteins in the NCBI/Uniprot database, for example, several ribosomal subunit proteins. It remains to be seen if these are spurious effector predictions, or a true reflection of their importance for virulence in wheat.

To gauge an approximate idea of the proportion of EV proteins involved in different cellular processes, proteins were assigned putative functional annotations based on predicted domains and sequence identity to characterised proteins in the Uniprot and NCBI non-redundant protein databases (Fig. 3, Additional file 2: Table S2). Based on this categorisation, EVs were distinct from the SS fraction in their protein cargo. Over 50% of SS proteins were uncharacterised proteins compared to 7% of EV proteins (Additional file 2: Tables S2, S3). Approximately a quarter of EV proteins assigned a functional description were putatively associated with sugar, lipid and other metabolic processes (24.3%) and 18.6% involved in amino acid metabolism and protein homeostasis/metabolism. This included putative heat-shock proteins (Hsp60, Hsp82, Hsp90 co-chaperone), a protein disulphide isomerase, and translational machinery such as translation elongation (EF-2, EF-1-gamma, EF-3) and initiation factors. Similarly, ribosomal subunit proteins constituted 15.7% of EV proteins. The proportion of proteins in these categories were comparatively low in SS samples. Interestingly, of the 30 proteins identified in both SS and EV fractions, most were associated with carbohydrate and lipid metabolism (23.3%) or carbohydrate hydrolytic activity (20%), or were uncharacterised (36.7%) (Fig. 3, Additional file 2: Table S3).

Given the membranous nature of EVs we expected a proportion of proteins to be membrane associated: 6.7% of proteins were putatively membrane associated and included ABC transporters and other ATPase domain-containing proteins. Proteins involved with endo- and exocytosis or the endomembrane system (3.3% of proteins) included clathrin, a putative t-SNARE protein, an ADP-ribsoylation factor and a Sar1 GTPase-like protein. Actin, tubulin and fimbrin were among 7 proteins (3.3%) associated with the cytoskeleton. Proteins in these categories were largely absent from the SS fraction, with the exception of a GPI-anchored uncharacterised protein.

### *Z. tritici* EV proteins overlap with proteins identified in other fungal EVs

**Fig. 4:**
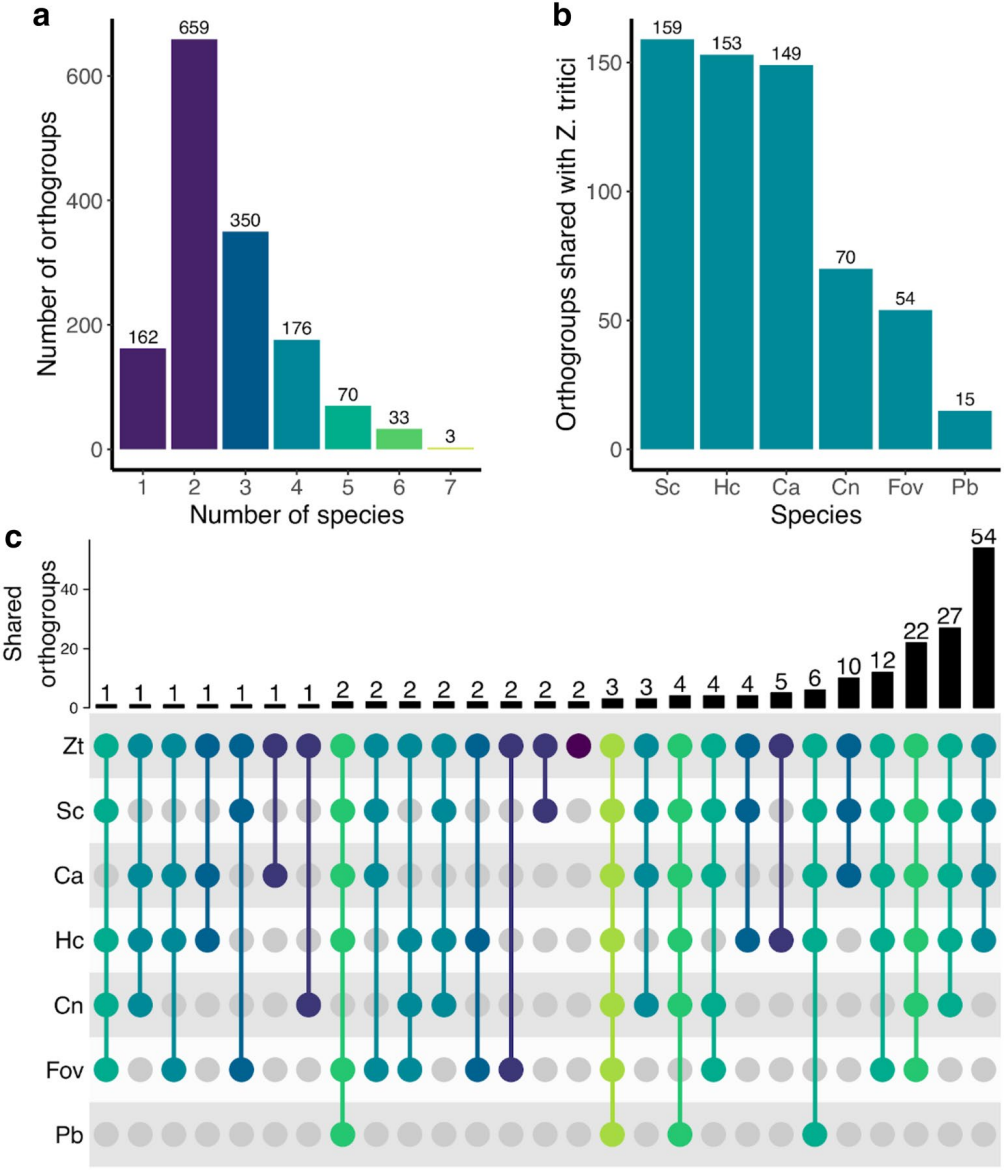
*Z. tritici* EV proteins group with EV proteins from other fungi based on sequence similarity. The fungi compared in the plot are *S. cerevisiae* (*Sc*), *H. capsulatum* (*Hc*), *C. albicans* (*Ca*), *C. neoformans* (*Cn*), *F. oxysporum* f. sp. *vasinfectum* (*Fov*) and *P. brasiliensis* (*Pb*). Proteins previously identified in fungal EVs were grouped into orthologous groups using Orthofinder; only intersections including *Z. tritici* were represented in the plot. **a** shows the number of orthogroups shared by all species; for example, 3 orthogroups are shared by all seven species. **b** Bar plot showing the number of orthogroups shared by *Z. tritici* and each fungal species. The intersection of *Z. tritici* orthogroups with those of the other fungi is represented with an Upset plot, shown in **c**. The bottom panel plots the intersections of orthogroups from each species. Each row represents a species and each column indicates which combination of species share *x* number of orthogroups. Dark, connecting dots indicate which species in a column share orthogroups. The bar plot at the top represents the size of the intersection, or how many orthogroups are shared by each combination of species. For example, 48 orthogroups are shared by *Z. tritici* (*Zt*) and *Sc, Hc* and *Ca*, while two orthogroups are species-specific to *Zt*

We looked for similar proteins in the cargos of other fungal EVs to determine the overlap of *Z. tritici* EVs with other fungi. We used the sequences of fungal EV proteins from previous proteomic studies of the human pathogenic fungi *C. albicans, C. neoformans*, *H. capsulatum* and *Paracoccidioides brasiliensis*; the cotton pathogen *Fov*; and *S. cerevisiae* (total n = 6648). The datasets and articles referred to are summarised in the additional files (Additional file 2: Table S5). 77.8% of these proteins were assigned to 1453 orthogroups (OGs), with the majority of OGs (88.9%) containing proteins from more than two species (Fig. 4a). Only 3 OGs included similar EV proteins from all 7 species (Fig. 4a, c). These consisted of peptidyl-prolyl cis–trans isomerase proteins (OG9), plasma membrane ATPases (OG12) and ADP-ribosylation factors (ARFs) (OG18), respectively, the latter including three ARFs characterised in yeast as proteins associated with vesicle trafficking (Additional file 2: Table S6). 95.5% of *Z. tritici* EV proteins were assigned to 177 OGs (12.2%), of which 2 were species-specific. *Z. tritici* EV proteins were most frequently grouped with similar proteins from *S. cerevisiae*, *C. albicans* and *Histoplasma,* though this is likely a reflection of the larger size of the EV proteomes currently defined for these fungi, compared to the *P. brasiliensis* or *Fov* proteomes (Fig. 4b, c). Orthogroups shared by *Z. tritici* and *Fov* exclusively were of interest, given their shared plant pathogenic lifestyles. Two groups of orthologous proteins were exclusive to these pathogens and consisted of subtilisin-like peptidases (OG1244) and peroxidase-catalase like proteins (OG765). Only 14 *Z. tritici* EV proteins were not assigned to an OG or grouped in a species-specific OG, suggesting significant similarity in the proteins identified in *Z. tritici* EVs and other fungal EVs characterised to date.

### The proposed fungal EV protein marker Sur7 is consistently detected in *Z. tritici* EVs

A recent comprehensive study of potential marker proteins for *Candida albicans* EVs showed that the Sur7-family members *Ca*Sur7 and Evp1 were promising markers for *C. albicans* EVs [40]. We found a putative *Ca*Sur7 homologue, here referred to as *Zt*Sur7, was consistently detected in *Z. tritici* EV samples from in vitro cultures (Additional file 2: Table S2). *Zt*Sur7 has a 28.3% pairwise sequence identity with *Ca*Sur7, a shared Sur7 protein domain (L12–I207) and four predicted TMDs with a similar topology to the *C. albicans* protein (Additional file 1: Figure S2a). This protein was not detected in secreted samples and was only detected in 2/5 cell lysate samples (CL), which we analysed alongside the *Z. tritici* EV samples (a list of proteins identified in CL samples is available in additional file 2, Table S7). This aligns with the criteria Dawson et al. used to identify this protein as a fungal EV protein marker in *C. albicans,* which was based on recommendations for marker proteins by the International Society of Extracellular Vesicles [40, 41]. Analysis of RNA-seq evidence from a previous study describing *Z. tritici* WAI332 gene expression *in planta* (Wang et al., under review) found the *Zt*Sur7 gene is expressed during infection of wheat at 9- and 14-days post infection (Additional file 1: Figure S2b).

Dawson et al. also proposed 7 negative marker proteins for *C. albicans* EVs. Negative markers are needed for confirming EV cargoes are distinct from the general contents of the cell or plasma membrane, as outlined by Thery et al. [40, 41]. We identified putative orthologous proteins in *Z. tritici* with a reciprocal best-hit blastp search. Orthologues of five of these *C. albicans* proteins—ABP1, APR1, Cyp1, LAP41 and LPD1—were identified in 80% of *Z. tritici* cell lysate (CL) samples (Additional file 2: Tables S7, S8). These proteins were not consistently identified in *Z. tritici* EVs. Together, this suggests Sur7 is an EV-associated protein and potential marker in *Z. tritici* as well as *C. albicans,* while *Z. tritici* proteins orthologous to the aforementioned negative protein markers, may also be applicable for studies of *Z. tritici* EVs in vitro.

## Discussion

While EVs from mammalian cell lines have been widely studied, an understanding of fungal EVs is lacking, particularly in the context of plant-pathogen interactions. Recently, the first report of EVs from the cotton pathogen *Fov* suggested its EVs may be implicated in pathogenesis [32]. We have now reported our study of EVs from an agronomically-significant wheat pathogen, *Z. tritici.* This is the first description and proteomic analysis of *Z. tritici* EVs secreted under in vitro conditions and it is anticipated this initial step will provide a foundation for future studies of *Z. tritici* EVs in the pathogen’s infection of wheat.

Our findings show *Z. tritici* produces small (< 100 nm) and some larger EVs (~ 100–300 nm) that are consistent with the EV populations isolated from other fungi [30]. Similarly, our proteomic analysis of *Z. tritici* EVs suggests the protein cargo broadly overlaps with existing fungal EVs proteomes, given the majority of proteins identified shared orthogroups with EV proteins from more than one fungal species. This observation, combined with the fact that the presence of EV particles appears reduced in heat-treated *Z. tritici* cultures—a result seen in other fungi [28, 42, 43]—consolidates the hypothesis that *Z. tritici* EVs are actively secreted by cells rather than being randomly-shed membranous artefacts [44]. To this end, *Z. tritici* EVs also contain some proteins consistently associated with mammalian EVs, including the cytoskeletal components actin and tubulin; proteins implicated in signal transduction such as 14-3-3 domain-containing proteins; clathrin, which is involved in mammalian EV biogenesis as well as clathrin-mediated endocytosis; and heat-shock proteins, including Hsp70 family and Hsp90 proteins [21, 45, 46]. Taken together, the overlap of *Z. tritici* EV protein cargo with that of other fungal EVs and mammalian EVs provides confidence these are similar to the EVs released by other fungi.

We identified 210 *Z. tritici* EV proteins that were present in at least 4/5 biological replicates. This mirrors the number of proteins identified in earlier proteomic studies of EVs from *C. neoformans, C. albicans* and *H. capsulatum*, which range from ~ 50 to 200 proteins [31, 42, 47]. It is significantly fewer than the number of proteins identified in most recent studies of EVs from *Fov* (482 EV proteins) [32]*, S. cerevisiae* (3133) [28] and *C. albicans* (from 729 to 1202 across different strains and morphologies in Zarnowski et al. [52] and Dawson et al. [40]). Based on the latter studies which identified > 400 proteins using stringent filtering thresholds and multiple biological replicates, the results presented here are likely an underestimation of the *Z. tritici* proteome. More comprehensive proteomic profiling of *Z. tritici* EVs from different fungal growth conditions or different strains may help to expand on the *Z. tritici* EV proteome defined here.

The proteins identified in *Z. tritici* EVs were grouped into functional categories based on sequence identity to characterised proteins and/or the presence of protein domains. The majority of proteins were putatively associated with cellular metabolic processes, particularly lipid and carbohydrate metabolism, and protein homeostasis (e.g. protein translation and folding) and amino acid metabolism. This broadly reflects the annotated functions associated with EV proteins in other fungi, particularly recent observations that fungal EVs are consistently enriched in proteins associated with protein and carbohydrate metabolism and are implicated in cell wall maintenance and remodelling [28, 37].

As with other fungal EVs, an Hsp70 family protein was present in *Z. tritici* EVs, with two others present in both EVs and the secreted fraction. Of the latter, one protein (evm.model.chr_10.464) had the highest sequence identity (86%) to the conserved Hsp70 chaperone in the model filamentous fungus *Neurospora crassa*. The presence of Hsp70 in EV and EV-free fractions was similarly observed in the human pathogen *P. brasiliensis*, and has speculated to be due to secretion via multiple pathways [48]. Hsp70 is a classical mammalian EV marker [45] and was suggested as a potential fungal EV marker (in combination with other markers) given its apparent ubiquity in fungal EVs [32, 37]. Whether the co-occurrence of Hsp70-like proteins in EV and the soluble supernatant in *Z. tritici* is the result of contaminated sample preparation, or because the protein is secreted by noncanonical and canonical secretory pathways remains to be seen. It suggests Hsp70 is unlikely to be a valid marker in this fungus until the nature of this co-occurrence is investigated.

Fungal EVs are implicated in virulence, evidenced by the presence of virulence-associated cargo and immunogenicity of EVs from some human pathogenic fungi. For example, EVs from *C. neoformans,* a pathogen of immunocompromised people, secretes EVs containing the major virulence proteins urease and laccase and the capsular polysaccharide glucuronoxylomannan (GXM) [31]. Another opportunistic human pathogen *C. albicans* secretes EVs carrying proteins associated with adhesion to host cells [37]. *C. albicans* EVs have also been shown to stimulate nitric oxide and cytokine production in immune cells and internalisation by bone-marrow derived macrophages [49]. Given this, EVs from human pathogenic fungi have been referred to as ‘virulence bags’ [31]. The role of EVs in the virulence of plant pathogens is unknown, although a recent analysis *Fov* EVs found they contained secondary metabolite (SM) biosynthetic proteins and an uncharacterised polyketide. This, combined with phytotoxic activity of the EVs in cotton and *Nicotiana benthamiana* leaves, led the authors to speculate on a role of *Fov* EVs in pathogenesis [32].

Similarly, we looked for proteins potentially implicated in pathogenesis but found no strong effector candidates or SM proteins associated with *Z. tritici* EVs. While some EV proteins were predicted to be effectors by the machine learning-based software EffectorP, most of these had sequence homology to highly conserved proteins, for example, 40S and 60S ribosomal proteins and a small COPII coat GTPase Sar1-like protein (involved in COPII complex required for endoplasmic reticulum transport vesicle assembly). Given fungal effectors are generally, though not always, uncharacterised with little sequence homology to other proteins, we suspect these predicted EV protein ‘effectors’ are likely false positives. In regards to the lack of SM-associated in *Z. tritici* EVs, it may be the presence of these in *Fov* EVs reflects the rich myco- and phytotoxin repertoire of *Fusarium* spp. [50], rather than being a ubiquitous feature of fungal EVs. This will only be elucidated with a broad analysis of proteomes and metabolomes of EVs from fungal phytopathogens. We also looked for proteases and CAzymes, given their importance in the lifestyle of plant pathogens, but these were also not prevalent in EVs relative to the secreted fraction.

We found a lack of obvious pathogenicity-associated proteins in in vitro-produced *Z. tritici* EVs, but this does not dismiss a possible role for EVs *in planta* during the pathogen’s infection of wheat. It is hard to speculate on this using these data given the EV protein cargo is likely very different *in planta.* The gene expression profiles of *Z. tritici in planta* and in vitro are distinct [5]. While some in vitro culture conditions like Fries medium can induce the expression of pathogenesis-associated genes in plant pathogenic fungi, the conditions we have used here cannot effectively simulate those the pathogen would experience *in planta.* This is particularly significant as mammalian EV cargoes typically reflect the state of the cell of origin and are influenced by changes in the cellular environment [51]. Similarly, the protein composition of fungal EVs has been shown to vary depending on the morphology of the cells and nutrients available in the environment. For example, protein cargo was distinct when EVs from the biofilm and planktonic form of *C. albicans* were compared [52], while the nutrient composition of culture media was found to influence EV release and protein composition in *H. capsulatum* [53]. *Z. tritici* grows as blastospores, a yeast-like form, in vitro but switches to hyphal growth during colonisation of the wheat apoplast [54]. Hyphal growth can be induced in cultures under nutrient-limited conditions and is linked to transcriptional changes, such as the increased expression of putative effectors, that reflect the *in planta* expression of virulence-associated genes [55]. A comparison of EVs from hyphal and blastospore morphologies in vitro may provide more insight into the proteins associated with EVs *in planta.* It is likely only studies of EVs isolated from the wheat apoplast during *Z. tritici* infection of susceptible and resistant cultivars that will reveal a role for EVs in pathogenesis.

Our understanding of the biological function of fungal EVs has been hampered by the lack of fungal specific EV markers. Markers are an essential quality control for EVs and can be used for immunoaffinity-based purification or labelling [41]. Dawson et al. [40] recently defined a set of potential positive and negative protein markers for *C. albicans* EVs, including the fungal specific transmembrane protein Sur7. Sur7 has been characterised as a plasma membrane protein in *C. albicans* and *S. cerevisiae* that localises to defined microdomains known as the membrane compartment of Can1 (MCC) and is required for proper cell wall and plasma membrane composition and organisation [56–58]. In this study, a Sur7 homologue (*Zt*Sur7) was consistently identified in *Z. tritici* EVs, suggesting its use as an EV marker may be applicable to other fungi. The validity of Sur7 proteins as an EV marker for fungal plant pathogens in the context of infection will depend on Sur7 being present in EVs *in planta.* That the *ZtSur7* gene is expressed *in planta* at early and late infection timepoints is promising, but further studies are needed to confirm the protein is loaded into EVs in this context. Given the fungal specific nature of this protein, it may provide a valuable resource for the separation of wheat- and *Z. tritici*-derived EVs from the apoplast, in combination with plant EV markers PEN1 and TET8, described by Cai et al. [23] and Rutter and Innes [25].

## Conclusions

In summary, we have reported the first evidence of EVs produced by the devastating wheat pathogen, *Z. tritici.* The morphology and protein composition of these EVs reflect that of previously characterised fungal EVs. While EVs have been shown to have a role in the virulence of human pathogens, their contribution to the lifestyle of plant pathogens is still unknown. It is tempting to speculate about the role of EVs in the *Z. tritici*—wheat interaction: are they a vehicle for the non-canonical secretion of effectors to the apoplast and/or host cells? Are EVs taken up by wheat cells and if so, how? Are *Z. tritici* EVs involved in microbial competition in the host? Further study of EVs in *Z. tritici*, using tools like the marker Sur7, will advance our knowledge of the disease lifecycle of this cryptic, apoplastic wheat pathogen and more broadly, our understanding of noncanonical secretion mechanisms in plant pathogens.

## Materials and methods

### Fungal growth conditions

*Z. tritici* isolate WAI332 was grown from glycerol stocks on yeast sucrose agar (YSA) for –4 days at 22 °C under a 12-h light/dark cycle. Cells scraped from agar medium were used to inoculate 100 mL of Fries 3 broth in 250 mL conical flasks to a final concentration of 2 × 10^5^ blastospores/mL and cultured for 72-h, shaking at 140 rpm (22 °C; 12-h light/dark cycle). At 72 h culture OD_600_ was measured to confirm growth between replicate cultures was consistent.

### EV isolation by differential ultracentrifugation (DUC)

EVs were isolated from broth cultures using methods adapted from Thery et al. and Rodrigues et al. [59, 60]. *Z. tritici* cells were removed from broth cultures by centrifugation at 4500×*g* for 25 min in a benchtop centrifuge. Culture supernatant was centrifuged at 15,000×*g* (4 °C) for 45 min in an Avanti J series High Performance centrifuge (Beckman coulter) with a JA 14.50 rotor to remove cell debris. Supernatant was carefully removed and passed through a 0.45 µm MF-Millipore membrane filter using vacuum filtration. EVs were pelleted from the culture filtrate by ultracentrifugation for 75 min at 100,000×*g* and 4 °C in open-top Ultra Clear 38.5 mL tubes (Beckman coulter, 344058) using an SW 32 Ti swinging bucket rotor and Optima XPN ultracentrifuge (Beckman Coulter). Supernatant was removed completely and the EV pellet washed with 1X phosphate buffered saline (PBS), pH 7.4 (filter sterilised with 0.22 µm membrane), by ultracentrifugation at 100,000×*g* (75 min, 4 °C). The EV pellet was resuspended in 50 to 100 µL of 1X PBS, pH 7.4 and protein content measured using the Qubit™ protein assay kit. EV samples were flash-frozen with LN_2_ and stored at − 80 °C. This process was repeated with a Fries3-only control to confirm EV-like particles were not an artefact of the growth medium.

### Heat-treatment of *Z. tritici* broth cultures

To test if EVs were a product of dead or dying cells cultures of *Z. tritici* in Fries 3 medium (inoculated to a final concentration of 2 × 10^5^ blastospores/mL) were grown for 72 h and the OD_600_ measured. Cells were pelleted gently by centrifugation (3000×*g*, 4 °C, 10 min), washed three times with fresh culture medium and heated at 55 °C for 1 h. Cells were pelleted, washed once and resuspended in 100 mL of fresh Fries 3 medium. A small aliquot of heat-treated cells was plated on YSA to confirm cells were not viable. Heat-treated cultures were incubated for 72 h, alongside untreated *Z. tritici* cultures, prepared as described above. At 72 h EVs were isolated from heat-treated and untreated cultures.

### Transmission electron microscopy (TEM)

EV samples were negatively stained for TEM imaging. Carbon-coated copper 200 mesh TEM grids were glow discharged (45 s; 15 milliamps) and incubated with 3 µL of EV sample for 1 min. Excess sample was blotted from grids with Whatman^®^ grade 1 qualitative filter paper (GE Healthcare). Grids were briefly washed by floating—sample-side down—on drops of distilled water, blotted and floated on 20 µL drops of 2% uranyl acetate for 45 s. Excess stain was blotted and grids dried before visualising with a Hitachi HA7100 transmission electron microscope, operated at 100 kV. All microscopy work was conducted at the Advanced Imaging Precinct of Microscopy Australia at The Australian National University.

### Nanoparticle tracking analysis

Size distribution and particle concentration of EV samples were determined using a NanoSight NS300 fitted with a blue laser (405 nm) with NanoSight NTA software version 3.3 (Malvern Panalytical). EV samples were diluted 50–200 times using 1X PBS, pH 7.4 filtered with a 0.02 µm pore size syringe filter (GE Healthcare). Samples were diluted so less than 100 trackable particles were detected in each video frame. Samples were injected into the flow cell at a flow rate of 40 units. Six 30 s captures were taken in triplicate for each sample, with a camera level of 12–14. For video analysis a detection threshold of 10 was used, with the minimum tracking distance set to ‘Auto’. The flow cell was flushed with filtered 1X PBS pH 7.4 between samples, until no light scattering was detected. The size distributions generated by the NTA v3.0 software were averaged over technical replicates and plotted using ggplot2 in R [61].

### Bottom-up proteomics using LC–MS/MS

In preparation for LC/MS–MS, EV samples were pelleted and washed with 1X PBS pH 7.4 using ultracentrifugation (100,000×*g*, 75 min, 4 °C) and resuspended in 100 µL of 1X PBS pH 7. A volume of EVs equivalent to 4 µg of protein was disrupted with sodium dodecyl sulfate (SDS) at a final concentration of 1% and gentle pipetting. Peptides for LC/MS–MS were prepared following a method designed by Dr. Adam Carroll (Joint Mass Spectrometry Facility, The Research School of Chemistry, The Australian National University). Briefly, EV proteins were reduced with a final concentration of 10 mM dithiothreitol (DTT) and alkylated with 30 mM iodoacetamide (IAA). SDS was removed using a salty acetone precipitation: ice-cold acetone and 30 mM NaCl (final concentration) was used to pellet proteins by centrifugation. Pellets were washed with ice-cold acetone and resuspended in ammonium bicarbonate using sonication. 160 ng of MS-grade trypsin (Pierce™, ThermoFisher Scientific) was used to digest proteins overnight at 37 °C. Excess ammonium bicarbonate was evaporated and the peptides resuspended in 0.1% (v/v) formic acid (FA). Non-ionic detergents and salts were removed using strong cation exchange ZipTip_scx_ pipette tips (Merck Millipore) following the manufacturer’s instructions. Peptide eluent from ZipTip elution was evaporated and the peptides resuspended in 0.1% FA for analysis by LC–MS/MS.

To allow qualitative presence/absence comparison be made with EV samples, soluble secreted protein (SS) and cell lysate samples (CL) were also prepared for LC–MS/MS following the described procedure. For the SS samples, the supernatant from ultracentrifugation of culture filtrate at 100,000×*g* was collected and centrifuged twice at 120,000×*g* for 2 h to remove residual EVs and insoluble debris. Cell lysate samples were prepared from the cell pellets of culture used for EV isolation. Cell pellets were washed twice with 1X PBS pH 7.4 before grinding tissue in LN_2_ with a mortar and pestle and solubilising proteins in total protein buffer (20 mM Hepes–NaOH pH 7.4, 150 mM NaCl, 5 mM EDTA, 3% SDS, 1 mM PMSF, 2 mM DTT). Soluble supernatant and CL samples were prepared as above for LC–MS/MS except the digested peptides of CL samples were passed through an Amicon centrifugal concentrator (MWCO 30 kDa; pre-washed with 50% methanol and dH_2_O) to remove residual nucleic acids, before removing excess ammonium bicarbonate as described above.

### LC–MS/MS

Typically, 10 µL of sample was injected into the LC–MS/MS system consisting of a Dionex UltiMate 3000 Rapid Separation LC (RSLC) system with an Orbitrap Fusion or Q Exactive Plus Orbitrap (Thermo Scientific). Peptides were trapped using an Acclaim PepMap100 C18 Trap column (Thermo Scientific) at a flow rate of 15 μL/min using 0.1% formic acid/2% acetonitrile in H_2_O (solvent A). Peptides were eluted from the trap column to an in-house packed column (75 µm inner diameter (ID) × 25 cm; 1.9 µm C18 media, Dr Maisch) with a pulled tip emitter. Peptides were eluted from the column using a linear 100-min gradient from 5% to 40% solvent B (0.1% formic acid/80% acetonitrile). Data was collected in positive mode and full spectra acquired from m/z 350 to 2000, with a resolution of 70,000. For MS/MS, the top 15 most intense precursor ions were fragmented and ionised, and MS/MS spectra with m/z 350–1750 and resolution 17,500 recorded.

### Analysis of LC/MS–MS data

SearchGUI v 3.3.17 was used to perform a high-sensitivity peptide search of raw MS/MS data files against a database of predicted protein sequences from *Z. tritici* WAI332 gene models (n = 15,019, (Wang et al., *in review*), with a target-decoy search strategy. SearchGUI was run with the search engines OMSSA, X! Tandem and MS-GF+, a peptide tolerance of 20 ppm, a fragment ion mass tolerance of 0.5 Da and maximum FDR threshold of 1% [62]. Other parameters were: oxidised methionine and N-terminal acetylation were allowed variable protein modifications; carbidomethylation of cysteines was an allowed fixed modification. Results were analysed using PeptideShaker v 1.16.44 [63]. Matches to contaminants, decoy sequences, ‘doubtful matches’ and matches with ≤ 2 unique peptides were excluded. Proteins detected in ≥ 2/3 replicates of at least one condition per experiment were included for analysis. Proteins were assigned functional annotations using InterProScan v5.0, run with default parameters [64] and blastP searches against the fungal nr and UniprotKB/Swissprot protein databases. SignalP 5.0 was used to identify proteins with canonical signal peptides [65]. Cazymes were identified with the dbCAN2 metaserver [66], proteases identified by searching sequences against the MEROPs database (release 11.0) [67] (non-peptidase homologue hits were excluded) and effectors predicted using EffectorP v 1.0 and v 2.0 [68]. Membrane-associated proteins were defined using TMHMM v 2.0 and Pred-GPI [69, 70].

### Grouping fungal EV proteins in orthogroups

Protein sequences from previous fungal EV studies were downloaded from Uniprot or Ensembl fungi and duplicate sequences within a species removed. Proteins were grouped into orthogroups using OrthoFinder v 2.3.11 [71], which was run with blastp for all-*vs*-all sequence searches (default parameters) and the multiple sequence alignment (msa) method for gene tree inference (using MAFFT and fasttree). Results were plotted using ggplot2 and the ComplexHeatmap packages in R.

## Supplementary information

**Additional file 1: Figure S1.** EVs are produced by *Z. tritici* cultured in multiple growth media and by at least two Australian strains of *Z. tritici*, WAI332 and WAI321. TEM data shows EVs isolated from *Z. tritici* WAI332 cultures grown in (A) Fries 3, (B) minimal medium and (C) potato dextrose broth. (D) TEM imaging of *Z. tritici* WAI321 EVs isolated from Fries 3 growth medium. All samples were stained with 2% uranyl acetate and visualised with a Hitachi H7100FA TEM at 100 kV. Images were cropped and scale bars added with ImageJ; images were not modified otherwise. **Figure S2.** A *Z. tritici* protein, *Zt*Sur7, is homologous to the proposed *C. albicans* EV marker, *Ca*Sur7. (a) An amino acid sequence alignment of *Zt*Sur7 with *Ca*Sur7, which share a SUR7 domain, 4 transmembrane domains (TMD) and putative cytoplasmic/extracellular domains. (b) Illumina RNA-seq reads (pink) from wheat infected with *Z. tritici* WAI332 aligned to the WAI332 genomic region encoding ZtSur7, showing expression of *Z. tritici Sur7* homologue *(ZtSur7) in planta* at 9 and 14 days post infection (dpi). Alignment coverage across the gene model is shown in grey in the top panel, while the gene model and a corresponding schematic are shown in blue.

**Additional file 2: Table S1.** Metadata for EV samples/fungal cultures presented in Fig. 1 and Fig. 2. **Table S2.** List of proteins identified in ≥ 4/5 biological replicate samples of *Z. tritici* WAI332 EVs. **Table S3.** List of proteins identified in ≥ 2/3 SS samples, with at least 2 unique peptides detected using LC–MS/MS. Proteins found in the SS samples and also in EVs are indicated. **Table S4.** List of *Z. tritici* WAI332 EV proteins predicted to be effectors by EffectorP. Proteins were included when predicted as effectors by both EffectorP v1.0 and v2.0. **Table S5a.** List of studies used to source fungal EV proteins to define orthogroups with Orthofinder. **Table S5b.** Summary of proteins used for Orthofinder analysis. **Table S6.** OrthoFinder output showing fungal EV proteins grouped by orthogroup (OG) and species. Uniprot-KB or ensembl protein names are followed by a species ‘code’ defined for Orthofinder. CAAL = *C. albicans*; CRNE = *C. neoformans*, FOTG = *F. oxysporum* f. sp. *vasinfectum*; HICA = *H. capsulatum;* PABR = *P. brasiliensis*; SACE = *S. cerevisiae*; ZYTR = *Z. tritici*. **Table S7.** List of proteins identified with ≥ 2 unique peptides in  ≥ 4/5 cell lysate (CL) samples (n = 533). **Table S8.** Potential *Z. tritici* WAI332 homologues for *C. albicans * EV marker proteins defined by Dawson et al., (2020). Homologous proteins were identified using reciprocal blastp searches of marker proteins against predicted proteins encoded in the *Z. tritici* WAI332 genome. **Table S9.** This data was generated by Orthofinder (v 2.3.11), using EV proteins described in previous fungal EV studies. Protein sequences were downloaded from Uniprot or Ensembl fungi and duplicate sequences removed within each species. Information on the studies listed and the proteins from each species is shown in Table S5.

## Data Availability

The datasets used and/or analysed during the current study are available from the corresponding author on reasonable request.
