# Supplementary material for: Extracellular vesicles from the apoplastic fungal wheat pathogen Zymoseptoria tritici
Source: Fungal Biol Biotechnol. 2020 Sep 18;7:13. doi: 10.1186/s40694-020-00103-2 (PMC7501697; doi:10.1186/s40694-020-00103-2)
Supplement: Supplementary file 1 — Additional file 1: Figure S1. EVs are produced by Z. tritici cultured in multiple growth media and by at least two Australian strains of Z. tritici, WAI332 and WAI321. TEM data shows EVs isolated from Z. tritici WAI332 cultures grown in (A) Fries 3, (B) minimal medium and (C) potato dextrose broth. (D) TEM imaging of Z. tritici WAI321 EVs isolated from Fries 3 growth medium. All samples were stained with 2% uranyl acetate and visualised with a Hitachi H7100FA TEM at 100 kV. Images were cropped and scale bars added with ImageJ; images were not modified otherwise. Figure S2. A Z. tritici protein, ZtSur7, is homologous to the proposed C. albicans EV marker, CaSur7. (a) An amino acid sequence alignment of ZtSur7 with CaSur7, which share a SUR7 domain, 4 transmembrane domains (TMD) and putative cytoplasmic/extracellular domains. (b) Illumina RNA-seq reads (pink) from wheat infected with Z. tritici WAI332 aligned to the WAI332 genomic region encoding ZtSur7, showing expression of Z. tritici Sur7 homologue (ZtSur7) in planta at 9 and 14 days post infection (dpi). Alignment coverage across the gene model is shown in grey in the top panel, while the gene model and a corresponding schematic are shown in blue. [file 40694_2020_103_MOESM1_ESM.pdf]

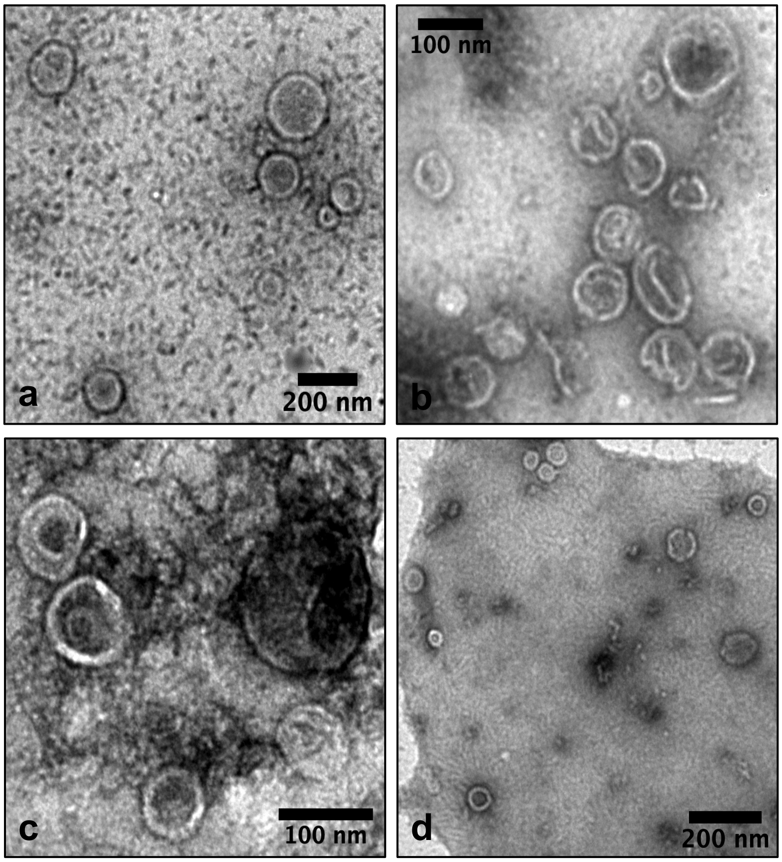


**Figure S1. EVs are produced by *Z. tritici* cultured in multiple growth media and by at least two Australian strains of *Z. tritici*, WAI332 and WAI321**. TEM data shows EVs isolated from *Z. tritici* WAI332 cultures grown in (A) Fries 3, (B) minimal medium and (C) potato dextrose broth. (D) TEM imaging of *Z. tritici* WAI321 EVs isolated from Fries 3 growth medium. All samples were stained with 2% uranyl acetate and visualised with a Hitachi H7100FA TEM at 100kV. Images were cropped and scale bars added with ImageJ; images were not otherwise modified.

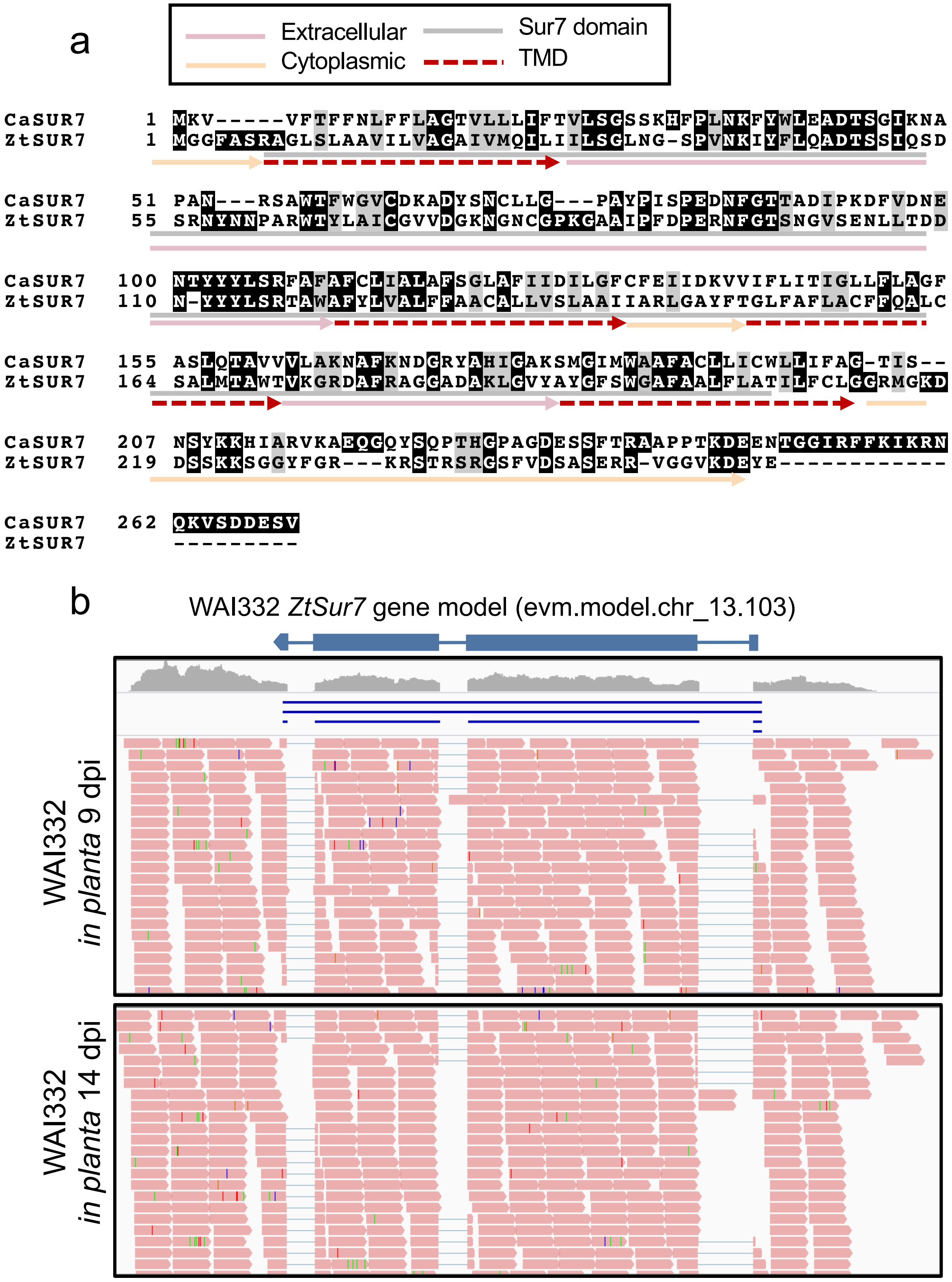


**Figure S2. A *Z. tritici* protein, *Zt*Sur7, is homologous to the proposed *C. albicans* EV marker, *Ca*Sur7.** (a) An amino acid sequence alignment of *Zt*Sur7 with *Ca*Sur7, which share a SUR7 domain, 4 transmembrane domains (TMD) and putative cytoplasmic/extracellular domains. (b) Illumina RNA-seq reads (pink) from wheat infected with *Z. tritici* WAI332 aligned to the WAI332 genomic region encoding ZtSur7, showing expression of *Z. tritici Sur7* homologue *(ZtSur7) in planta* at 9 and 14 days post infection (dpi). Alignment coverage across the gene model is shown in grey in the top panel, while the gene model and a corresponding schematic are shown in blue.
